# Efficient Removal of Micro-Sized Degradable PHBV Microplastics from Wastewater by a Functionalized Magnetic Nano Iron Oxides-Biochar Composite: Performance, Mechanisms, and Material Regeneration

**DOI:** 10.3390/nano15120915

**Published:** 2025-06-12

**Authors:** Huaguo Xia, Nini Duan, Beisi Song, Yuan Li, Hongbin Xu, Ying Geng, Xin Wang

**Affiliations:** 1 School of Ecology and Environment, Zhengzhou University, Zhengzhou 450001, China; xhgzhizhu@stu.zzu.edu.cn (H.X.); duannn@gs.zzu.edu.cn (N.D.); songbeisi@stu.zzu.edu.cn (B.S.); xuhongbin_gy@zzu.edu.cn (H.X.); gengying@zzu.edu.cn (Y.G.); 2 Engineering Research Center for Water Emergency Response of Henan Province, Zhengzhou 450001, China; 3 Research Institute of Frontier Science, Southwest Jiaotong University, Chengdu 610031, China; xin.wang@swjtu.edu.cn

**Keywords:** magnetic biochar, Poly(3-hydroxybutyrate-co-3-hydroxyvalerate), degradable microplastics, adsorption, magnetic separation

## Abstract

The co-occurrence of the synthesis of a functionalized magnetic nano iron oxides–biochar composite (MFe@BC) via impregnation–thermal pyrolysis and its use to remove micro-sized poly (3-hydroxybutyrate-co-3-hydroxyvalerate) (PHBV) microplastics from simulated wastewater was demonstrated in this study. The results showed that PHBV removal efficiency correlated positively with MFe@BC dosage, achieving an adsorption capacity of 13.14 mg/g and a removal efficiency of 98.53% at an optimal dosage of 1.5 g/L. Adsorption kinetics fit a pseudo-second-order model (R^2^ = 0.9999), and the isotherm followed the Langmuir model (R^2^ = 0.8440), yielding a theoretical maximum capacity of 31.96 mg/g. Characterization indicated chemisorption-driven monolayer adsorption via surface complexation and hydrogen bonding. Magnetic nano-iron transfer from MFe@BC to the PHBV surface imparted magnetic properties to PHBV, enabling synergistic adsorption and magnetic separation. Removal efficiency remained above 95% across pH 4–9 and COD 0–500 mg/L. Regeneration experiments indicated that the MFe@BC showed robust reusability, maintaining >92% PHBV removal efficiency after four adsorption–regeneration cycles. The results of this study may provide a feasible pathway for PHBV microplastic removal from secondary effluent, indicating that MFe@BC prepared in this study can be used for the removal of PHBV microplastics in a wide range of water bodies.

## 1. Introduction

Since their commercialisation in the mid-20th century, synthetic plastics have revolutionised technological progress, yet their environmental legacy remains profoundly challenging. Global plastic production currently exceeds 350 million tonnes annually [1], of which an estimated 76% are ultimately discarded into landfills or natural ecosystems due to deficiencies in recycling infrastructure [2,3]. While conventional plastics are designed for durability, their persistent synthetic polymers gradually fragment through photolytic, mechanical, and biological weathering, generating microplastics (MPs)—an emerging particulate pollutant that persists in various environmental media and causes pollution problems [4]. The environmental mobility of MPs is amplified by their small particle size and durability, enabling long-range transport via aquatic currents and atmospheric circulation [5]. Smaller-sized (micron or nano-sized) MPs can even be transferred to plant tissues, internal animal organs, and even cross cellular barriers to accumulate in human blood vessels and brains [6]. This ubiquity raises significant ecotoxicological concerns, since MPs in living organisms could result in impaired feeding behaviour, histological damage to digestive organs, reduced survival rates across species, reproductive damage, and even brain lesions [7]. Furthermore, MPs exhibit a propensity to act as vectors for co-occurring pollutants. Their hydrophobic surfaces and long-lasting feature facilitate the sorption of multiple pollutants such as antibiotics, heavy metals, and hydrophobic organic contaminants, thereby altering the environmental fate and bioavailability of these hazardous substances [8,9,10].

The substitution of conventional non-degradable plastics with biodegradable plastics that can be mineralised in situ by microorganisms represents a promising strategy for addressing plastic pollution. However, the mineralization of biodegradable plastics under environmental conditions is usually incomplete [11]. Even more, due to their inherently weak mechanical stability and bio-degradability, biodegradable plastics exhibit heightened propensity for rapid MPs generation and secondary metabolite release [12], thereby exacerbating risks of MPs accumulation in ecosystems.

Among biodegradable polymers, polyhydroxyalkanoates (PHA), an aliphatic polyester, have become one of the most representative biodegradable polymers due to their competitive biodegradability and low production costs [13,14]. Poly(3-hydroxybutyrate-co-3-hydroxyvalerate) (PHBV) is one of the most representative varieties of PHA families, which has been extensively utilised in agricultural mulching, pharmaceutical delivery systems, textiles, and sustainable packaging industries [15,16]. However, particularly concerning is that PHBV undergoes incomplete degradation under natural conditions: field studies report <1.6 ± 0.2% mass loss in PHBV films after 6-month incubation in surface water, with residual fragments undergoing progressive embrittlement and microplastic formation [17]. Existing studies demonstrated that, in natural environments (such as soil or aquatic systems), the degradation efficiency of PHBV was significantly lower than that observed under industrial composting conditions. Micron- and nanoscale particles continued to be generated over time, resulting in sustained bioavailability and associated environmental exposure risks. Under soil conditions, PHBV microplastics were degraded by only 1.5–5% within eight weeks. Furthermore, as the concentration of PHBV increased, nitrogen source limitation was induced, which led to a further reduction in degradation efficiency and subsequently altered the structure of the soil microbial community and disrupted nutrient cycling [18]. Such persistent PHBV-derived MPs demonstrate ecotoxicological hazards, including causing a dose-dependent reduction in plant growth and damaged foliar metabolic function in the soil–plant system [19] and causing acute toxic effects on marine plankton in the water system [20]. Recent studies have highlighted that biodegradable microplastics (BMPs) can pose ecological risks that are as high as—or even higher than—those of conventional microplastics (CMPs) when they co-exist with other pollutants. For example, Ye et al. reviewed the environmental fate and ecotoxicity of CMPs versus BMPs and pointed out that polymers such as PLA and PHA frequently fail to fully degrade in natural water and soil. Instead, they fragment into micro- and nano-sized particles that exhibit a higher sorption affinity for trace contaminants (e.g., heavy metals, pesticides) compared to CMPs, leading to similar or greater toxic effects on organisms (algae, nematodes, and fish) [21]. Therefore, although PHBV was classified as a biodegradable material under industrial composting conditions, its persistence and potential ecotoxicological effects in natural environments rendered it a critical target for research focused on the development and assessment of degradable microplastic removal technologies, necessitating the implementation of targeted interventions for their effective removal from environmental matrices.

Wastewater treatment plants (WWTPs) represent a critical pathway for microplastic (MP) dissemination into aquatic ecosystems. Although conventional treatment stages (e.g., activated sludge units, primary sedimentation) achieve partial MPs retention, a substantial proportion of MPs (usually on microsize grade) evades removal due to operational limitations in tertiary treatments (e.g., membrane filtration, advanced oxidation processes) and are subsequently discharged via effluent streams [22]. Global estimates indicate that municipal WWTPs release approximately 3 and 23 billion (an average of 13 billion) MPs daily into receiving water bodies, constituting a major anthropogenic pollution vector [23]. Consequently, efficient MPs removal technologies for secondary effluents are imperative to mitigate environmental risks.

Current MP removal strategies include adsorption, coagulation–flocculation, catalytic oxidation, etc [24]. Compared with other technologies, adsorption is prioritised due to its operational simplicity, cost-effectiveness, and scalability, particularly for submicron MPs [25]. Commonly used adsorbents for microplastic removal include biochar-based, sponge-based, metal-based, etc. [25,26]. Among them, biochar—a pyrolytic carbonaceous material—has emerged as a promising adsorbent owing to its tuneable surface porosity, chemical stability (ash content <20%), and sustainable sourcing from agricultural waste [27]. However, conventional biochar exhibits intrinsic limitations such as low MPs adsorption capacity, poor reusability, and challenges in post-adsorption recovery from sludge matrices [28].

Recent advances in functionalised magnetic biochar design have compensated for these shortcomings to some extent [29]. Metal-modified variants, such as iron-containing modified biochar, demonstrate enhanced MPs affinity due to the formation of chemical bonds between the modified biochar and MPs and the increased adsorption active sites [30]. Furthermore, the magnetism from the loaded iron components enables rapid solid–liquid separation by magnetic separation methods, which allows the simultaneous removal of MPs from sewage and sludge and also facilitates the recycling of adsorbents [31].

In this study, a functionalized magnetic iron oxide–biochar composite (MFe@BC) was synthesised via an impregnation–pyrolysis protocol using straw-derived biochar (BC) as the substrate, with ferrous chloride tetrahydrate (FeCl_2_·4H_2_O) and ferric chloride hexahydrate (FeCl_3_·6H_2_O) as precursors to confer enhanced magnetic separation capabilities. The adsorption efficacy of MFe@BC towards micro-sized PHBV MPs (<5 μm) in simulated effluent was systematically evaluated by varying critical operational parameters, including adsorbent dosage, solution pH, reaction time, PHBV concentrations, and co-existed COD. The regeneration performance of MFe@BC was also investigated. Adsorption mechanisms were elucidated through kinetic and thermodynamic analyses, complemented by comprehensive material characterisation (SEM-EDS, FTIR, XRD) of MFe@BC and PHBV substrates pre- and post-adsorption. These findings establish MFe@BC as a technically robust and recyclable adsorbent for mitigating biodegradable microplastic pollution in wastewater effluents, offering a scalable strategy for tertiary treatment system integration.

## 2. Materials and Methods

### 2.1. Quantification of PHBV MPs

Pristine Poly(3-hydroxybutyrate-co-3-hydroxyvalerate) (PHBV) MPs (5 μm, white powder) was purchased from NatureWorks Co., Ltd. (Plymouth, MN, USA). The PHBV MPs used in this study underwent a biodegradation procedure to simulate its characteristic in the actual WWTPs effluents: 25 g of pristine PHBV was added to a 1 L beaker containing 1000 mL of filtered effluent from the biochemical units of WWTPs. Then, the PHBV suspension was subjected to magnetic stirring (480 rpm; Model ZNCL-BS; Henan Aibot Technology Development Co., Ltd., Zhengzhou, China) within a light-proofed glass beaker. The ageing duration was set to 24 h according to the upper limit of conventional hydraulic retention time of sewage in WWTPs. The aged PHBV MPs were filtrated, washed by deionized water for some times, and dried at 25 °C by a constant-temperature drying oven (Model DHG-9071A; Shanghai Jinghong Experimental Equipment Co., Ltd., Shanghai, China) for further use.

A PHBV calibration curve was established via UV–Vis spectrophotometry. The optimal analytical wavelength was determined by performing a full-spectrum scan on the stock solution using a spectrophotometer (Model UV-6300; Shanghai Mepda Instrument Co., Ltd., Shanghai, China), revealing a characteristic absorption maximum at 243 nm. To prepare the PHBV standard solutions (1, 2, 3, 5, and 10 mg/L), PHBV was added in 50 mL deionized water within an amber stoppered flask and ultrasonic treated for 30 min at 25 ± 1 °C to achieve homogenization. Triplicate absorbance measurements of the prepared PHBV standard solutions at 243 nm were acquired using quartz cuvettes with deionized water serving as the blank. A linear regression model (y = 0.0138x − 0.0004, R^2^ = 0.9999) correlating absorbance (*y*-axis) with PHBV concentration (*x*-axis, mg/L) was derived.

To minimize the influence of experimental errors, three parallel samples and a blank control (a control sample with MFe@BC in absence of PHBV) were set up for all of the adsorption experiments. And the quantification of PHBV in the experimental sample was achieved by subtracting the absorbance of the blank control from the absorbance of the test experimental sample. The removal efficiency (R) and adsorption capacity (q_e_) of MFe@BC on PHBV were calculated using Equations (1) and (2).

(1) $$Removal\;rate(R)=\frac{\left(C_{0}-C_{t}\right)}{C_{0}}\times 100\%$$

(2) $$Adsorption\;capacity(q_{e})=\frac{\left(C_{0}-C_{t}\right)\times V}{W}$$

where C_0_ is the concentration of PHBV solution at zero h; C_t_ is the concentration of PHBV solution at t h; q_e_ (mg/g) is the mass of PHBV adsorbed; V is the volume of solution used in each experiment; and W is the mass of absorbent used in each experiment.

### 2.2. Preparation of MFe@BC

Nano iron oxides–biochar composite (MFe@BC) was synthesised via an impregnation–pyrolysis protocol using straw-derived biochar (BC) as the substrate, with ferrous chloride tetrahydrate (FeCl_2_·4H_2_O) and ferric chloride hexahydrate (FeCl_3_·6H_2_O) as precursors [30]. The detailed preparation methods of MFe@BC are given in the Supporting Information, Text S1. The obtained MFe@BC was stored at room temperature for further use.

### 2.3. Batch Adsorption Experiments

For all batch adsorption experiments, samples were placed in a constant-temperature shaking shaker (Model QYC-200; Shanghai Xinmiao Medical Device Manufacturing Co., Ltd., Shanghai, China) at 150 rpm, 25 °C for the designed adsorption time. After the adsorption, a neodymium magnet (60 mm long, 10 mm wide, 5 mm thick, made of neodymium iron boron) was wrapped in a sealing film and placed in the sample with tweezers. The magnet was removed after 10 s, thereby separating the MFe@BC adsorbed with PHBV from the sample, and the absorbance of the remaining solution was measured at 243 nm for the calculation of the removal efficiency and adsorption capacity. To minimize the experimental errors, a blank control (a control sample with MFe@BC in the absence of PHBV) was set up for all of the adsorption experiments. All samples were run in triplicate.

#### 2.3.1. Effects of MFe@BC Dosage

To evaluate the effect of MFe@BC dosage on PHBV adsorption, MFe@BC (0.1, 0.5, 1.0, 1.5, 2.0 g/L) was accurately precisely and added to the solution of PHBV MPs (20 mg/L, pH = 7). Adsorption was carried out in a constant temperature shaker at a speed of 150 rpm, a temperature of 25 °C, and an oscillation of 24 h.

#### 2.3.2. Effect of Solution pH on Adsorption

To assess the effect of solution pH on PHBV adsorption by MFe@BC, PHBV suspensions were adjusted to pH 4.0–9.0 using 0.1 mol/L HCl and 0.1 mol/L NaOH. The dosage of MFe@BC for each sample was set at 1.5 g/L. Adsorption lasted for 24 h. After adsorption, the removal efficiencies and adsorption capacities were calculated, and the pH condition that afforded the highest PHBV removal was identified and employed in all subsequent experiments.

#### 2.3.3. Adsorption Isotherms

In order to explore the effect of different initial concentrations on the adsorption of PHBV by MFe@BC, this study carried out adsorption isotherm experiments under the conditions of MFe@BC dosage of 1.5 g/L and pH = 7. The PHBV initial concentration range was 5–200 mg/L. Adsorption lasted for 24 h. To further clarify the adsorption mechanism, the adsorption isotherm data were fitted into the Langmuir isotherm model (3), the Freundlich isotherm model (4), and the Temkin isotherm model (5).

Langmuir isotherm model:

(3) $$q_{e}=\frac{q_{m}K_{L}C_{e}}{1+K_{L}C_{e}}$$

Freundlich isotherm model:

(4) $$q_{e}=K_{F}{C_{e}}^{1/n}$$

Temkin isotherm model:

(5) $$q_{e}=K_{T}lnC_{e}+K_{T}lnf$$

where q_e_ (mg/g) is the adsorption amount at adsorption equilibrium for different initial concentrations of downloaded iron oxide nanobiochar; q_m_ (mg/g) is the maximum adsorption amount fitted by the isotherm model; K_L_ (L/mg), K_F_ (L/g), and n are the conventional parameters in the Langmuir and Freundlich model related to the maximum adsorption amount; C_e_ (mg/L) is the concentration of PHBV solution at adsorption equilibrium; K_T_ (mg/g) is the slope in the Temkin model; and f is the activity (or fugacity) of the adsorbate in the Temkin model.

#### 2.3.4. Adsorption Kinetics

For kinetic study, 1.5 g/L of MFe@BC was added to a 20 mg/L PHBV solution (pH 7.0), and the reaction was allowed to proceed for 0.083, 0.250, 0.500, 1.000, 2.000, 4.000, 16.000, 24.000, 32.000, 40.000, and 48.000 h. Each mixture was agitated in a thermostatic orbital shaker at 150 rpm and 25 °C. At the designated time, samples were withdrawn, and the PHBV removal efficiency and adsorption capacity of MFe@BC were determined. To further clarify the adsorption mechanism, the adsorption kinetic data were fitted into the pseudo-first-order model (6), pseudo-second-order model (7), and the Elovich model (8).

Pseudo-first-order kinetic modelling:

(6) $$q_{t}=q_{e}(1-e^{-K_{1}\cdot t})$$

Pseudo-second-order kinetic modelling:

(7) $$q_{t}=\frac{q_{e}2K_{2}t}{q_{e}K_{2}t+1}$$

The Elovich model:

(8) $$q_{t}=\frac{1}{\beta }ln(\alpha \beta t+1)$$

where $q_{e}$ (mg/g) is the adsorption capacity at equilibrium; $q_{t}$ (mg/g) is the adsorption capacity at contact time (t); K_1_ (h^−1^) and K_2_ (g/(mg·h)) are the rate constants for pseudo-first-order and pseudo-second-order kinetic reactions; α (g/(mg·h)) is the initial adsorption rate; and β (mg/g) is the desorption constant.

#### 2.3.5. Effect of Co-Existed COD on PHBV Adsorption

The actual water body contains a large amount of organic matter, which is an important factor affecting adsorption [32,33]. To evaluate the effect of the co-existing organic matter on PHBV adsorption, the concentration of the co-existed chemical oxygen demand (COD) was set at 15~500 mg/L by adding a certain amount of glucose (C_6_H_12_O_6_, AR) in PHBV solution (PHBC concentration = 20 mg/L). The pH of the mixture was adjusted to 7 and the MFe@BC dosage was set at 1.5 g/L. Adsorption lasted for 24 h. Glucose was selected as a model compound to represent dissolved organic matter (DOM) owing to its simple molecular structure, high aqueous solubility and reproducible behaviour during adsorption experiments. It was used to facilitate a controlled investigation of the fundamental interactions between MFe@BC composites and organic matter. Although the structural complexity inherent in natural organic matter (NOM), such as humic and fulvic acids, was not encompassed by glucose, it was employed as an initial probe for elucidating adsorption mechanisms. Subsequent research was planned to assess the adsorption performance of MFe@BC composites in the presence of more structurally complex NOM to more accurately simulate natural aquatic matrices [34].

#### 2.3.6. Characterization of PHBV and MFe@BC

The surface morphology and element distribution of MFe@BC, PHBV, and regenerated MFe@BC were determined by field emission scanning electron microscopy coupled to an energy dispersive spectrometer (SEM-EDS, ZEISS Sigma 360, Jena, Germany). The surface functional groups of involved materials were determined by the Fourier transform infrared spectrometer (FT-IR). The adsorption specific surface area of the material was determined by the BET specific surface area meter. The magnetization of MFe@BC was measured at 298 K as a function of the applied magnetic field (±20,000 Oe) using a vibrating sample magnetometer (VSM) to obtain its saturation magnetization (M_s_). The composition of the material was determined by X-ray diffraction (XRD), and the surface element composition and valence state of MFe@BC before and after adsorption were determined by X-ray photoelectron spectrometer (XPS).

#### 2.3.7. Regeneration of MFe@BC

In order to investigate the reuse efficiency of MFe@BC, this study carried out regeneration experiments at an MFe@BC dosage of 1.5 g/L and a pH of 7.0. Before each adsorption–regeneration cycle, deionized water was adjusted to pH 7.0 with 0.1 mol/L NaOH and 0.1 mol/L HCl. For each cycle, 1.6 mg of PHBV and 0.12 g of MFe@BC were introduced into 100 mL centrifuge tubes, adding 80 mL of pH-adjusted solution. Triplicate samples and corresponding blank controls (containing MFe@BC only) were prepared. The tubes were agitated at 150 rpm and 25 °C in a thermostatic orbital shaker for 24 h. Thereafter, the MFe@BC was subjected to magnetic separation, and the absorbance at 243 nm was used to determine the removal efficiency and adsorption amount of PHBV.

The spent MFe@BC was regenerated and reused for four successive cycles as follows. First, it was dried at 80 °C for 24 h in an oven, then calcined in a tubular furnace at 400 °C for 2 h (heating rate 10 °C/min^−1^). The calcined material was dispersed in deionized water, stirred, and allowed to settle, and the supernatant was decanted and filtered three times. The retained solids were washed with anhydrous ethanol (stirred briefly and left to stand for 5 min), filtered, washed twice more with ethanol, and rinsed with deionized water. After a final drying at 80 °C for 24 h, the regenerated MFe@BC was employed in the next cycle. Upon completion of four cycles, the surface morphology of MFe@BC was examined by field-emission scanning electron microscopy. The contents of Fe^2+^ and total iron in MFe@BC and regenerated MFe@BC were measured by 1,10-phenanthroline spectrophotometry (powder pillow package) and FerroVer method (powder pillow package).

## 3. Results and Discussion

### 3.1. Characterization of MFe@BC

**Figure 1:**
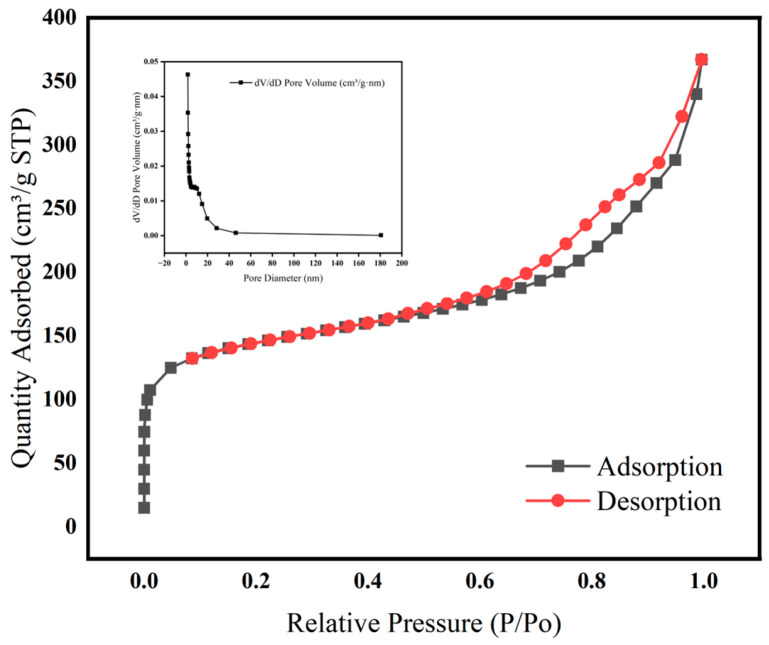
Adsorption–desorption curves of MFe@BC.

As shown in Figure 1, a high BET specific surface area was exhibited by MFe@BC, which was particularly crucial for its application in adsorption. The curve in the high-pressure region of the figure tended to be stable, reflecting that the total pore volume can characterize the internal pore properties of the material more accurately. The micropores contribute the most to determine the adsorption capacity, and a higher total pore volume means that the material has a larger holding space, which is advantageous in the adsorption of macromolecules or porous mass transfer and belongs to the range of small and medium-sized mesopores, which is very suitable for the adsorption of small molecules or oligomers [35]. The principal pore size distributions of the material were revealed by the dV/dD versus pore size (D) distribution curves in the embedded plots: the distribution peaks were mainly concentrated in the mesoporous region, indicating that MFe@BC was dominated by mesoporous structures, which was highly favourable for applications requiring high reactive mass transfer and molecular diffusion; additionally, it was indicated by some low-pore-size regions that the samples also contained a certain percentage of microporous structures.

**Figure 2:**
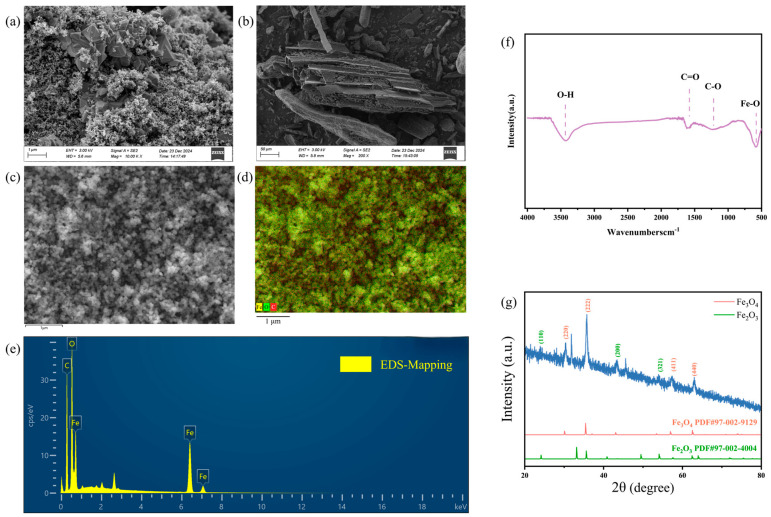
SEM image showing the surface morphology of MFe@BC: (**a**) 1 μm; (**b**) 50 μm; (**c**) Electron image of MFe@BC. (**d**) Layered image of MFe@BC. (**e**) EDS elemental mapping spectrum showing the total distribution of C, O, and Fe. (**f**) FTIR of MFe@BC. (**g**) XRD of MFe@BC.

The porous, granular-stacked morphology with pronounced surface roughness of nano-sized MFe@BC was given in Figure 2a,b, indicative of an increased specific surface area of MFe@BC that provided additional sites for adsorption and catalytic reactions [36]. At low magnification, an interconnected porous network was evident. At high magnification, numerous nanoscale particle clusters and a few flaky or needle-like crystals were observed to be uniformly deposited on the MFe@BC substrate. These iron oxides were firmly bound to the char matrix via mechanical occlusion and chemical adsorption without discernible agglomeration or peeling. The rough, porous surface significantly increased the specific surface area and diffusion pathways, thereby facilitating the transport and capture of adsorbates. Concurrently, the loaded iron-oxide nanostructures introduced additional chemically active sites and conferred a strong magnetic response, thus enabling one-step magnetic separation following adsorption.

As shown in Figure 2c–e, the EDS spectra revealed prominent peaks of Fe, O, and C, confirming that iron (Fe) and oxygen (O) are present in the composite and that the carbon matrix (C) is retained. While the Fe signals indicate incorporation of iron-based components—suggesting the presence of magnetically responsive phases—the specific identification of Fe_3_O_4_ or Fe_2_O_3_ crystalline phases is based on XRD analysis (see Figure 2g). The detection of O also implies that hydroxyl, carboxyl, and other oxygen-containing functional groups may exist on the biochar surface, which together with the iron species can synergistically enhance microplastic adsorption. In addition, in Table S1, the average value of the mass fraction of Fe at three independent measurement points is 55.36 ± 0.10 wt%, and the standard deviation is very small, indicating that the sample has a highly consistent Fe distribution at different points. The corresponding atomic fraction is 22.10 ± 0.10 At%, which further proves the repeatability of the material preparation process [37]. In addition to the point analysis, Figure 2c,d shows the spatial distribution of Fe, O, and C on the surface of the composite material. The results show that Fe is evenly distributed on the biochar matrix without obvious agglomeration, which further supports the repeatability of the preparation method (EDS data of MFe@BC are given in Supporting Information Table S1).

As shown in Figure 2f, the O–H vibrational peak at 3420.08 cm^−1^ indicated that the material possessed abundant oxygen-containing functional groups, which enhanced its hydrophilicity and interaction with polar pollutants [38]. The C=O peak at 1599.50 cm^−1^ suggested that these groups provided active sites during adsorption. The C–O peak at 1223.61 cm^−1^ implied that oxygen-containing moieties—such as alcohols, phenols, and ethers—were retained or generated in the MFe@BC during pyrolysis, exerting a significant influence. An Fe-O absorption appearing around 580.52 cm^−1^ confirms the incorporation of iron oxide species in the composite. According to XRD results, the iron oxide species could be Fe_3_O_4_ or Fe_2_O_3_ (see Figure 2g). Collectively, the O-H, C=O, and C-O signals demonstrated that the modified biochar retained a rich surface chemical structure, while the Fe-O absorption further corroborated the presence of the magnetic iron phase, thereby facilitating facile separation and reuse.

Figure 2g indicated a broad diffraction hump at around 2θ = 20°–30°, corresponding to the amorphous carbon skeleton of MFe@BC. In contrast to the sharp diffraction peaks of crystalline materials, MFe@BC only showed a broad and slow diffraction background due to its relatively disordered structure. The sharp diffraction peaks in the figure indicated that some degree of iron oxide crystal structure was present in the material, suggesting that not all of it was in amorphous form. The diffraction peaks observed at 2θ values of 30.1°, 35.5°, 43.1°, 53.4°, and 57.0° were assigned to the (220), (311), (400), (511), and (440) planes of magnetite (Fe_3_O_4_), indicating the presence of magnetic iron oxide phases within the composite. In addition, weaker but discernible peaks at 2θ = 24.2°, 33.2°, and 35.6° were attributed to the (012), (104), and (110) planes of haematite (α-Fe_2_O_3_), confirming the presence of non-magnetic Fe_2_O_3_ in the sample. Overall, the X-ray diffraction patterns demonstrated that both ferromagnetic Fe_3_O_4_ and antiferromagnetic α-Fe_2_O_3_ phases were incorporated into the biochar matrix during the synthesis of MFe@BC [39].

The magnetic properties of the MFe@BC composite and the biochar (BC) precursor were investigated by vibrating sample magnetometry (VSM). As illustrated in Figure S1, the magnetic behaviour of the MFe@BC composite was studied at 298 K over a field range of ±20,000 Oe. The resulting hysteresis loop exhibited negligible coercivity and remanence, indicating superparamagnetic character. A saturation magnetization (M_s_) of approximately 42 emu/g was recorded at an applied field of 20,000 Oe. By comparison, pristine biochar (BC) displayed no appreciable magnetisation under identical conditions. These findings demonstrate that the incorporation of iron oxide nanoparticles endowed the composite with a sufficiently high magnetic response to enable rapid and efficient magnetic separation from aqueous media.

To evaluate the effect of iron impregnation and thermal treatment on the physicochemical properties of biochar, pristine BC, and the Fe-impregnated composite (MFe@BC) were analysed by BET and EDS. The nitrogen adsorption–desorption isotherms and pore size distribution curves of BC (Figure S2g) revealed that the specific surface area was measured to be 1330.7 m^2^/g, the total pore volume was determined as 0.7612 cm^3^/g, and the average pore diameter was calculated to be 6.4046 nm (Table S2). Following the impregnation of iron and subsequent thermal treatment, MFe@BC was found to exhibit a significantly diminished specific surface area of 525.1 m^2^/g, a reduced pore volume of 0.5677 cm^3^/g, and an average pore diameter of 4.3243 nm (Table 1). Such reductions were attributed to partial pore blockage by iron oxide nanoparticles and slight structural collapse during calcination. Analysis by SEM-EDS further corroborated the successful incorporation of iron into the biochar matrix. Pristine BC was found to contain 84.58 wt% C and 15.29 wt% O, with a trace amount of Fe (0.13 wt%) (Table S3). In contrast, MFe@BC comprised 33.90 wt% C, 10.74 wt% O, and 55.36 wt% Fe (Table S1). The substantial increase in Fe content confirmed that the impregnation step effectively decorated the biochar surface with iron oxide species.

The pore structure, surface functional groups, morphology, and phase composition of the MFe@BC were comprehensively characterized by BET, SEM-EDS, FTIR, XRD, and VSM analyses. It was demonstrated that the material was endowed with a high specific surface area, abundant oxygen-containing functional groups, and controllable magnetic properties, conferring excellent adsorption capacity and facile magnetic separability. Consequently, the material exhibited a broad potential for application in wastewater treatment.

### 3.2. Effect of MFe@BC Dosage on PHBV Adsorption

**Figure 3:**
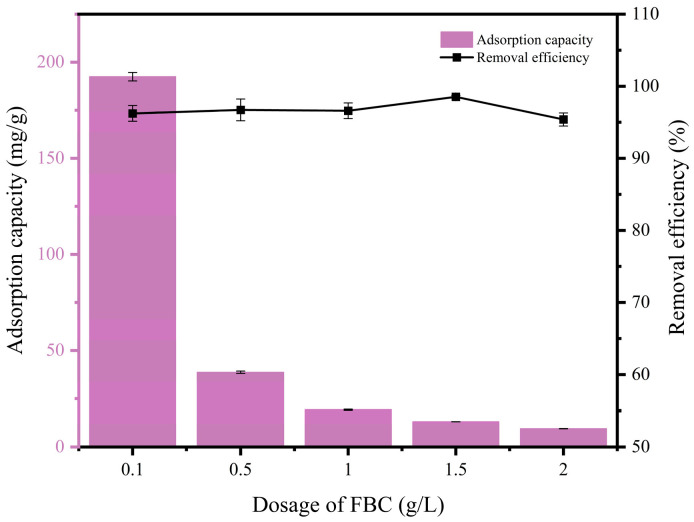
Effects of MFe@BC dosage on adsorption.

As shown in Figure 3, with the increase of MFe@BC dosage, the removal efficiency tended to rise, peaked at the dosage of 1.5 g/L (reached 98.53% with an adsorption capacity of 13.14 mg/g), and then declined with the increasing dosage. The removal efficiency was consistently above 95.39% over the experimental dosage range. As the dosage increased, the overall active sites increased and could contact with more PHBV molecules, and the removal efficiency was somewhat improved. However, when the dosage was increased beyond a certain threshold, the PHBV concentration (initial or residual) in the system could no longer occupy additional active sites simultaneously, and adsorption equilibrium was attained prematurely, resulting in no further significant improvement, or even a slight decrease, in removal efficiency [40,41]. At high dosages, MFe@BC particles were observed to aggregate, reducing the specific surface area and the effectiveness of the available active sites. Furthermore, although the diffusion distance for PHBV molecules to reach the active sites was shortened (due to the increased particle density), the effective surface area of each individual particle was reduced, thus increasing mass transfer resistance and causing a minor decline in removal efficiency at higher dosages [42]. With increasing MFe@BC dosage, the adsorption capacity per unit mass decreased from a maximum of 192.46 mg/g to 9.54 mg/g. This was attributed to the fact that, although the overall PHBV removal by the system was increased (as reflected in the removal efficiency), the adsorption capacity per unit mass declined owing to the greater amount of adsorbent. Considering both removal efficiency and unit adsorption capacity, a dosage of 1.5 g/L was selected as optimal, since a high removal rate of 98.53% was achieved while a relatively high adsorption capacity (13.14 mg/g) was maintained, thereby balancing economy and adsorbent utilisation efficiency.

Under identical experimental conditions (adsorbent dosage of 1.5 g/L and initial microplastic concentration of 20 mg/L), the MFe@BC composite achieved a removal efficiency of 98.53% and an adsorption capacity of 13.14 mg/g. For comparison, Shi et al. reported that magnetic biochar (CCBC) removed approximately 76.80% of microplastics, corresponding to an adsorption capacity of ~12.0 mg/g [43]. Similarly, Babalar et al. prepared a PVA-coated magnetic activated biochar–zeolite composite (PVA-MABC), which attained a removal efficiency of 95.2% for polystyrene microplastics (25 mg/L) with a 1.5 g/L adsorbent dosage, yielding an adsorption capacity of 11.5 mg/g [44]. In comparison with these studies, the MFe@BC composite exhibited both a higher removal efficiency and a slightly greater adsorption capacity under comparable conditions, thereby demonstrating its superior performance in the removal of PHBV microplastics from aqueous environments.

Previous studies found that pure biochar (Pinus roxburghii-derived BC) was shown to achieve an efficiency of 78% for 20 m/L PVC microplastics at pH 10 after 6 h of contact, corresponding to a maximum adsorption capacity of 131.5 mg/g in wastewater [45]. Pure Fe_3_O_4_/Fe_2_O_3_ nanoparticles (NPs) were also reported to attain efficiencies of approximately 70–95% for 50–200 m/L organic dye systems within 1–2 h, with adsorption capacities of 50–200 mg/g; however, owing to their three-dimensional morphology and tendency to agglomerate, their actual performance towards microplastics >100 nm was expected to be much lower than that observed for dye removal [46,47]. By contrast, the MFe@BC composite material (at a dosage of 1.5 g/L) was demonstrated to achieve an efficiency of 98.53% under 20 mg L^−1^ PHBV conditions, corresponding to an adsorption capacity of 13.14 mg/g; when the dosage was increased to 2.5 g/L, the adsorption capacity per unit mass decreased to 9.54 mg/g, yet the removal efficiency remained above 95.39%. It was therefore evident that MFe@BC combined the adsorption capacity of biochar with the polar and coordination-active sites of iron oxide, thereby markedly improving the removal efficiency and practical adsorption capacity for PHBV microplastics, whilst enabling rapid magnetic recovery, and outperforming both the pure BC and pure NP systems.

### 3.3. Effect of Solution pH on PLA Adsorption by MFe@BC

**Figure 4:**
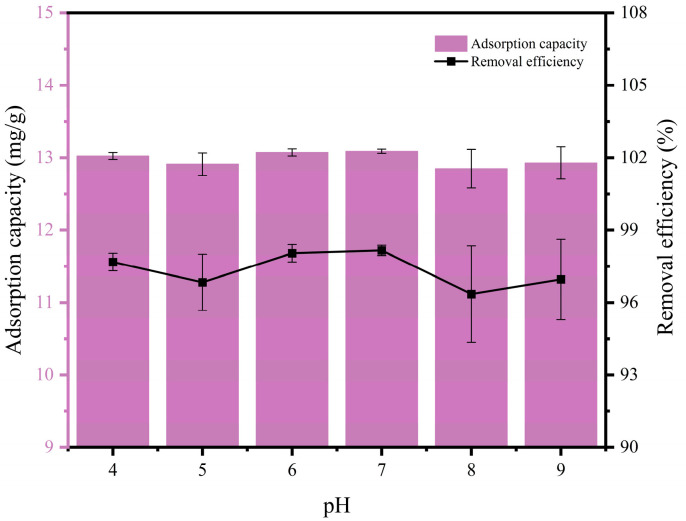
Effect of solution pH on adsorption of PHBV by MFe@BC.

Figure 4 demonstrated that the removal efficiency of MFe@BC for PHBV was effectively pH-independent and exceeded 95% across a broad pH range (4–9). The removal efficiency (98.04–98.16%) and adsorption capacity (13.07 mg/g–13.09 mg/g) reached relatively high values around pH 6–7 and peaked at neutral or slightly acidic conditions, indicating that the adsorption of PHBV by MFe@BC was most favourable in this interval. MFe@BC contained a large amount of oxygen-containing functional groups that were protonated or dissociated at different pH levels, resulting in corresponding changes in surface charge properties [48]. Hydrogen bonding and π–π interactions may also be established between MFe@BC and PHBV [49]. The strengths of these interactions depend on the protonation states of surface functional groups at varying pH levels, thereby influencing overall adsorption efficiency.

### 3.4. Adsorption Isotherms

**Figure 5:**
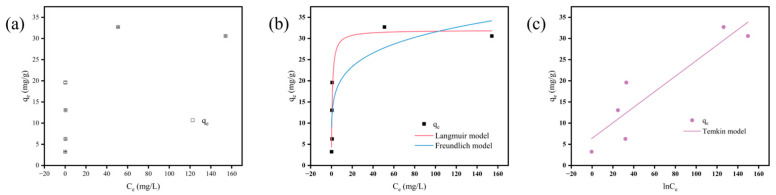
(**a**) Effect of initial PHBV concentration on the adsorption of PHBV by MFe@BC; (**b**) fitting curves of the Langmuir and Freundlich models; (**c**) fitting curves of the Temkin models.

The experimental data were fitted into Langmuir, Freundlich, and Temkin models, and the fitting parameters are given in Table 2. The Results showed that the Langmuir model was better fitted, with a goodness of fit *R*_2_ = 0.8440. The Langmuir model exhibited a steep initial ascent in adsorption capacity (q_e_) at low equilibrium concentrations (C_e_), followed by a distinct plateau in higher C_e_ regions (Figure 5b), suggesting that the adsorption process of PHBV on MFe@BC may be more inclined to monolayer homogeneous chemisorption, and the adsorption sites on the surface are homogeneous and equivalent. When equivalent adsorption sites on MFe@BC are progressively occupied, precluding further PHBV adsorption beyond monolayer coverage. Freundlich isotherm revealed a rapid q_e_ increase at low C_e_, moderating at elevated concentrations but deviating in the saturation regime (Figure 5b). The Freundlich constant K_f_ = 13.35, reflecting a substantial adsorption capacity of MFe@BC towards PHBV, while a 1/n value > 1 indicated a pronounced surface energy heterogeneity. This suggests preferential occupation of high-energy active sites (such as groups that can form chemical bonds with PHBV) during early adsorption stages, with limited contribution from lower-energy regions. The Temkin model demonstrated marked deviation from linearity across all C_e_ ranges, negating strong electrostatic interactions as a dominant mechanism (Figure 5c) [50]. Combined with Langmuir/Freundlich results, this further confirms that adsorption of PHBV by MFBC is more of a monolayer chemisorption mediated by surface complexation or chemical bonding, while physical interactions (e.g., hydrogen bonding or van der Waals forces) might also contribute to the adsorption.

### 3.5. Adsorption Kinetics

**Figure 6:**
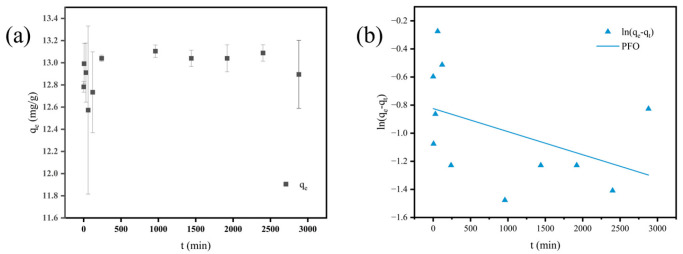

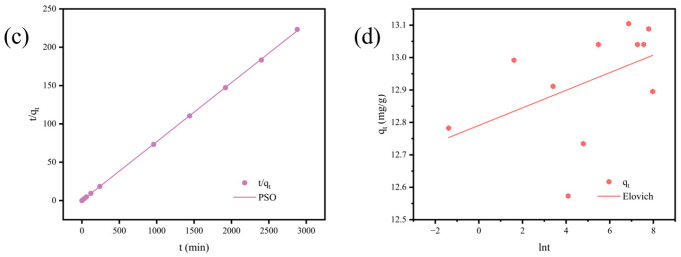
(**a**) Adsorption capacity of MFe@BC for PHBV at different reaction times; (**b**) fitting curves of the PFO models; (**c**) fitting curves of the PSO models; (**d**) fitting curves of the Elovich models.

The adsorption amount of PHBV on MFe@BC increased rapidly with the adsorption time, reaching 13.04 mg/g at the 240th minute of adsorption, and the removal efficiency reached 97.80%. It could be observed from Figure 6a that after 240 min of adsorption, the adsorption almost reached equilibrium, and the removal efficiency was stable at more than 97%. The kinetic data were fitted into PFO, PSO, and Elovich models, and the fitting parameters are given in Table 3. The pseudo-first-order model exhibited limited applicability (*R*^2^ = 0.1177), with a flat profile beyond 100 min failing to capture the gradually increased adsorption capacity (Figure 6b), confirming that the PFO model is not the most suitable description, which may only play a specific reference value in the early stage of adsorption.

In contrast, the PSO model demonstrated exceptional linearity (*R*^2^ = 0.9999), indicating that the PHBV adsorption onto MFe@BC could be chemisorption dominated by strong interactions such as ligand interaction and chemical bonding. As PHBV molecules gradually occupy the surface sites of MFe@BC, the available sites decrease and the adsorption rate decays in a quadratic manner. This aligns with Langmuir isotherm findings, corroborating relatively strong monolayer adsorption governed by strong interfacial interactions between PHBV and MFe@BC [51]. The *R*^2^ of the Elovich model reaches 0.1404, the scatter points deviate seriously from the straight line, and the parameter α is abnormally large and has no physical meaning. The Elovich equation assumes that the surface energy distribution is highly heterogeneous and the adsorption rate decreases logarithmically, which is inconsistent with the experimental situation.

### 3.6. Effect of Co-Existing COD on PHBV Adsorption

**Figure 7:**
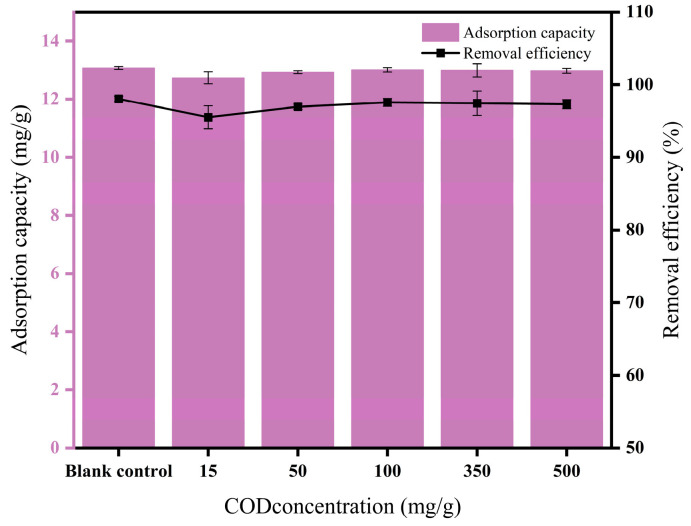
Effect of co-existed COD concentration on adsorption of PHBV by MFe@BC.

It could be observed from Figure 7 that the adsorption of PHBV by FBC was rarely affected by the coexisting COD, and the removal efficiency consistently exceeded 95% over the experimental COD concentrations (0–500 mg/L). When the COD concentration was 15 mg/L, the adsorption and removal efficiency decreased to some extent. In comparison, as the COD concentration increased to 50 mg/L and 100 mg/L, the adsorption and removal efficiency rebounded, indicating that a moderate amount of organic matter might promote the adsorption of PHBV. At high COD concentrations of 350 mg/L and 500 mg/L, the adsorption amount remained stable, and the removal efficiency decreased slightly, indicating that the competitive effect of high organic matter on adsorption was enhanced, and the removal of PHBV was inhibited. This suggested that the competitive adsorption between COD and PHBV on the surface of the MFe@BC inhibited the adsorption of PHBV, especially at low COD concentrations. However, a higher coexisted COD concentration (100–500 mg/L) promoted the change of polarity or hydrophobicity of the biochar surface [52], which enhanced the PHBV adsorption performance. In summary, coexisting COD had little effect on PHBV adsorption, and the removal efficiency of PHBV by MFe@BC could still be maintained at more than 97% under the COD concentration range of 100–500 mg/L, indicating that MFe@BC prepared in this study can be used for the removal of PHBV microplastics in a wide range of water bodies (e.g., secondary effluents, high-concentration organic wastewaters, etc.).

The selection of glucose as a model DOM provides insights into the basic adsorption behaviour of MFe@BC composites. However, it is important to recognize that natural waters contain a diverse range of organic compounds, including humic substances, proteins, and polysaccharides, which may interact differently with microplastics and adsorbents. Studies have shown that the presence of NOM can significantly influence the adsorption of microplastics and associated contaminants. Therefore, future investigations should incorporate various types of NOM to comprehensively assess the performance of MFe@BC composites in real-world scenarios [53].

### 3.7. Potential Mechanism of PHBV Adsorption by MFe@BC

**Figure 8:**
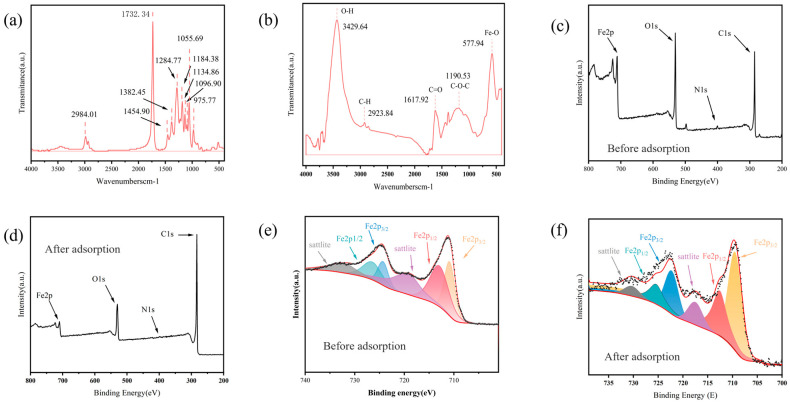
(**a**) FTIR of PHBV; (**b**) FTIR of MFe@BC after adsorption; (**c**) XPS full spectrum of MFe@BC before adsorption; (**d**) XPS full spectrum of MFe@BC after adsorption; (**e**) XPS fine spectra of Fe2 of MFe@BC before adsorption; (**f**) XPS fine spectra of Fe of MFe@BC before adsorption.

As shown in Figure 8a, the pronounced absorption band at 1732.34 cm^−1^ was ascribed to the C=O stretching vibration of the ester moiety, thereby confirming the presence of ester linkages. The band at 2984.01 cm^−1^ corresponded to C-H stretching vibrations in the alkyl chains (-CH_2_ and -CH_3_), reflecting the backbone architecture of the butyrate and valerate units [38]. The appearance of multiple C-H vibrational bands between 3000 and 2800 cm^−1^ further substantiated the chemical environment of these alkyl chains. Moreover, C-O-C asymmetric stretching vibrations in the 1500–1000 cm^−1^ region, together with C-H bending and C-O vibrations near 1500 cm^−1^, corroborated the integrity of the ester bonds. No O-H stretching bands were detected in the 3200–3600 cm^−1^ range, indicating that hydroxyl groups had been fully esterified or that the sample remained devoid of moisture. Likewise, the absence of C=C stretching bands in the 1600–1680 cm^−1^ region suggested that the material had neither undergone significant degradation nor oxidation, thus confirming its high purity (The FTIR data of PHBV and the FTIR data of MFe@BC after adsorption are given in Supporting Information Table S4).

The interaction mechanism between MFe@BC and PHBV can be better revealed by comparing the results in Figure 2f and Figure 8b. Following adsorption, the surface functional groups of MFe@BC were observed to have undergone distinct shifts. The hydroxyl (-OH) stretching vibration band was red-shifted from 3420.08 cm^−1^ to 3429.64 cm^−1^, suggesting that -OH groups had participated in hydrogen-bond formation during PHBV adsorption. The carbonyl (C=O) absorption peak shifted from 1599.50 cm^−1^ to 1617.92 cm^−1^, accompanied by an increase in band intensity, indicating that the carbonyl moieties on MFe@BC had interacted with the polar regions of the PHBV molecule. New bands at 2923.84 cm^−1^ (C-H stretching) and 1190.53 cm^−1^ (C-O-C asymmetric stretching), characteristic of PHBV, appeared after adsorption, thereby confirming the successful binding of PHBV to the biochar surface. Moreover, the Fe-O band exhibited a blue shift of 3 cm^−1^ (from 580.52 cm^−1^ to 577.94 cm^−1^), implying an increase in dipole moment or electron density transfer within the Fe-O moiety. This shift demonstrated that the iron-oxide magnetic structure had remained intact and had participated in complexation or chemical bonding throughout the adsorption process.

The adsorption mechanisms were further elucidated by comparative analysis of the peak position and relative contribution in the O1s, N1s, and Fe2p XPS spectra of MFe@BC before and after adsorption (Figure 8 and Figure S3, Table 4). The C1s deconvolution spectra resolved three distinct peaks corresponding to aromatic C-C (284.8 eV), alkyl ether C-O (286.3 eV), and ketonic carbonyl C=O (288.1 eV) functionalities (Figure S3a). After adsorption, a marked increase in the C-O (21.17% to 5.88%) and C=O (5.84% to 10.08%) components was observed, accompanied by a slight redshift (shift by 0.80 eV) of the C-C peak, indicating the coordination between the ester/ether oxygen groups of PHBV and the hydroxyl/iron groups of MFe@BC (Figure S3a,d), as well as the hydrogen bonding interaction. The Fe2p_3/2_ and Fe2p_1/2_ doublets with shake-up satellites maintained the positions and spin-orbit splitting characteristic of magnetite (Fe_3_O_4_) before and after adsorption (Figure 8e,f), confirming the retention of crystalline magnetic phases. In particular, the emergent N1s subpeaks at 400.1–401.8 eV after adsorption suggested the interfacial interactions between the ester groups of PHBV and N-containing groups of MFe@BC via hydrogen bonding or dipole interactions (Figure S3e). Furthermore, the percentages of Fe-O and C-O in the O1s spectra showed significant increases after adsorption, suggesting that the coordinated bonding between Fe oxides and the organic oxygen groups of PHBV occurred (Figure S3f) [54] (The XPS spectra of C1s, N1s, and O1s of MFe@BC before and after adsorption are given in Figure S2 of the Supporting Information).

SEM-EDS of PHBV and MFe@BC after adsorption are shown in the Supporting Information (Figure S4, Table S5). The morphology and elemental distribution of the nano-sized adsorbed PHBV MPs and the nano-sized adsorbed PHBV in the adsorbent were compared, the results showed that the PHBV before adsorption showed a typical rod-like or granular microstructure with a smooth surface and clear boundaries [55,56], whereas the surface of the PHBV after adsorption was covered by nanoscale rough agglomerates (Figure S4i), and the high-magnification scanning electron microscopy images (Figure S4j) showed that the pristine PHBV particles were tightly embedded in the porous structure of the charcoal substrate, which confirmed that the PHBV could be stably adsorbed on the MFe@BC. The EDS mapping results further revealed the adsorption mechanism (Figure S4g,h,o,p, Table S5): there was almost no Fe in the PHBV before adsorption, while a marked increase in Fe content (>2.53%) was observed for the adsorbed PHBV, with spatial co-localisation of Fe signals and surface-adherent nanoparticles (Figure S4n). This suggests that iron was transferred from the magnetic iron oxides loaded on MFe@BC to the surface of PHBV during the adsorption process. This iron translocation might be attributed to coordination bonding between PHBV ester groups and Fe-O moieties on MFe@BC or the chemisorption and the precipitation of the leached iron onto PHBV surfaces, as corroborated by XPS results. Notably, the transferred iron species endowed PHBV with magnetic properties, enabling synergistic removal of PHBV through MFe@BC adsorption and high-gradient magnetic separation.

### 3.8. Regeneration of MFe@BC

**Figure 9:**
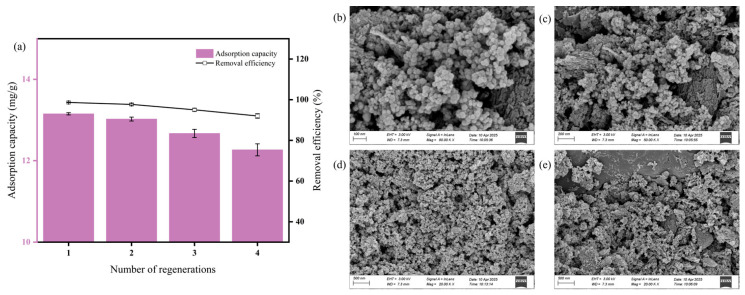
(**a**) Effect of the number of reclaims on the adsorption of PHBV by MFe@BC; SEM image showing the surface morphology of regenerated MFe@BC: (**b**) 100 nm; (**c**) 200 nm; (**d**) 500 nm; (**e**) 500 nm.

Figure 9a illustrated that removal efficiency and adsorption capacity declined with successive cycles, indicating a gradual diminution in performance; nevertheless, both metrics remained high overall. The reduced efficiency was attributed to incomplete decomposition or removal of PHBV during regeneration, which led to pore blockage, a decrease in accessible pore volume and diffusion pathways, and a corresponding loss of adsorption sites. After four cycles, the adsorption capacity remained at 12.267 mg/g and the removal efficiency exceeded 92%, demonstrating both the stability and recovery potential of MFe@BC. Furthermore, its magnetic separability was preserved after four adsorption–desorption cycles, thereby significantly reducing the cost of the adsorbent in practical applications. Even following repeated regeneration, MFe@BC continued to exhibit high adsorption capacity and removal efficiency, confirming its robust recycling potential.

As evidenced by the comparison of Figure 2a,b, Figure 9, and Figure S2a,b, following four PHBV adsorption–regeneration cycles, SEM analysis demonstrated that the prepared MFe@BC retained excellent morphological stability and cyclic robustness. In the nano-sized, the porous carbon framework bearing uniformly dispersed spherical and clustered nano iron oxides–biochar composites remained structurally intact, with no observable pore collapse or occlusion and only minimal nanoparticle agglomeration.

After leaching experiments, the unit mass leaching of MFe@BC was 5.85 mg/g, including 1.15 mg/g of Fe^2^⁺ and 4.70 mg/g of Fe^3^⁺, and the unit mass leaching of MFe@BC after four regeneration experiments was 10.83 mg/g, with Fe^2^⁺ and Fe^3^⁺ accounting for 4.04 mg/g and 6.79 mg/g. These results indicate that although a certain degree of iron loss will occur during repeated use, the composite material remains intact and retains its functionality. Although there is measurable Fe leaching under extremely acidic conditions, and the dissolution of MFe@BC after four regenerations is slightly increased compared with MFe@BC, if the Fe content of MFe@BC is above 50wt%, the corresponding Fe loss rate is only in the range of 1–3%.

Despite repeated regeneration, MFe@BC preserved a high specific surface area, abundant active sites, and strong magnetic responsiveness while maintaining material purity. These findings affirmed the durability and reusability of this nano iron oxides–biochar composite in the efficient adsorption and magnetic recovery of aqueous organic pollutants such as PHBV, underscoring its promising potential for multiple adsorption–regeneration operations in practical water-treatment processes.

## 4. Conclusions

In this study, a new-type functionalized magnetic iron oxide-biochar composite MFe@BC was synthesized and used to remove micro-sized PHBV microplastics in simulated wastewater. The results indicated that the removal efficiency of PHBV by MFe@BC could reach 98.53% under optimal conditions (pH = 7, adsorbent dosage of 1.5 g/L). Kinetic and isotherm studies revealed that PHBV adsorption obeyed the pseudo-second-order model (*R*^2^ = 0.9999) and the Langmuir model (*R*^2^ = 0.8440), suggesting that the adsorption was more inclined to the monolayer chemisorption. Characterization analysis demonstrated that adsorption was mainly driven by surface complexation and hydrogen bonding, accompanied by the magnetic separation induced by the transfer and recrystallization of dissolved Fe components of MFe@BC to the PHBV surface. The removal efficiency remained above 95% over a wide range of co-existed COD concentrations (0–500 mg/L^−1^) and pH (5–9). Notably, after four adsorption–regeneration cycles, MFe@BC still showed a high PHBV removal efficiency (exceeded 92 %), confirming the durability and reusability of MFe@BC. These findings establish MFe@BC as an efficient and durable adsorbent for the removal of PHBV MPs from aqueous environment, suggesting its significant potential for practical implementation in the remediation of MPs contaminated water.

## Figures and Tables

**Table 1:** BET data of MFe@BC.

Specific Surface Area	Porosity	Average Pore Size
525.1104 m^2^/g	0.567678 cm^3^/g	4.3243 nm

**Table 2:** Fitting parameters of the adsorption isotherm of PHBV by MFe@BC.

Model	Parameters		
Langmuir	q_e_	mg/g	1.26
	K_L_	L/mg	31.96
	*R* ^2^		0.8440
Freundlich	n		0.19
	K_F_	(mg/g)(L/mg)^1/n^	13.35
	*R* ^2^		0.7309
Temkin	lnf		3.71
	K_T_	mg/g	3.87
	*R* ^2^		0.7986

**Table 3:** Fitting parameters of adsorption kinetics of PHBV by MFe@BC.

Model	Parameters		
PFO	q_e_	mg/g	0.44
	K_1_	min^−1^	−1.64 × 10^−4^
	*R* ^2^		0.1177
PSO	q_e_	mg/g	12.99
	K_2_	g/(mg·min)	−0.06
	*R* ^2^		0.9999
Elovich	α	mg/(g·min)	1.80 × 10^203^
	β	g/mg	36.87
	*R* ^2^		0.1404

**Table 4:** XPS data of MFe@BC before and after adsorption.

		Before Adsorption		After Adsorption	
		Peak Position (eV)	Proportion (%)	Peak Position (eV)	Proportion (%)
C	C-C	283.86	72.99	283.06	84.03
	C-O-C	285.14	21.17	284.77	5.88
	O-C=O	287.84	5.84	286.96	10.08
Fe	Fe2p_1/2_	726.46	12.09	725.40	8.23
		712.92	27.48	712.60	18.93
	Fe2p_3/2_	724.28	8.79	722.28	18.11
		710.85	19.78	709.48	41.15
	sattlite	731.73	4.67	730.30	4.12
		718.93	22.53	717.50	9.47
N	Ta4p_3/2_	400.10	23.76	399.55	31.42
		399.04	41.58	398.78	30.27
	N1s	398.77	34.65	396.90	38.31
O	Organic C-O	532.05	51.02	532.00	38.76
	Metal oxide	530.31	25.51	530.24	38.76
	Metal carbonate	529.16	23.47	528.67	22.48

## Data Availability

Data are contained within the article and Supplementary Materials.

## Supplementary Materials

The following supporting information can be downloaded at: https://www.mdpi.com/article/10.3390/nano15120915/s1, Figure S1: VSM diagram of MFe@BC. Figure S2: SEM-EDS and BET of BC. Figure S3: XPS graphs of C1s, N1s, and O1s of MFe@BC before and after adsorption. Figure S4: SEM-EDS of PHBV and MFe@BC after adsorption. Table S1: EDS data of MFe@BC. Table S2: BET data of BC. Table S3: EDS data of BC. Table S4: FTIR of PHBV and FTIR data of MFe@BC after adsorption. Table S5: EDS data of PHBV and adsorbed MFe@BC.
