# Supplementary material for: Efficient Removal of Micro-Sized Degradable PHBV Microplastics from Wastewater by a Functionalized Magnetic Nano Iron Oxides-Biochar Composite: Performance, Mechanisms, and Material Regeneration
Source: Nanomaterials (Basel). 2025 Jun 12;15(12):915. doi: 10.3390/nano15120915 (PMC12196454; doi:10.3390/nano15120915)
Supplement: Supplementary file 1 [file nanomaterials-15-00915-s001.zip › nanomaterials-3666227-supplementary.pdf]

Efficient Removal of Micro-sized Degradable PHBV Microplastics from Wastewater by a Functionalized Magnetic Nano Iron Oxides-Biochar Composite: Performance, Mechanisms, and Material Regeneration

Huaguo Xia[1,2], Nini Duan[1,2], Beisi Song[1,2], Yuan Li[1,2*], Hongbin Xu[1,2],Ying Geng[1,2], Xin Wang[3]

1 School of Ecology and Environment, Zhengzhou University, Zhengzhou, Henan 450001, PR China

2 Engineering research center for water emergency response of Henan Province, Zhengzhou, 450001, China

3 Research Institute of Frontier Science, Southwest Jiaotong University, Chengdu, 610031, China

Supporting Information

TEXTs:

TEXT S1. Preparation method of MFe@BC

5.4 g $FeCl_2 \cdot 4H_2O$ and 4.0 g $FeCl_3 \cdot 6H_2O$ were dissolved in 100 mL deionized water and stirred at 480 rpm for 30 min. Then, 3.0 g BC was added and stirred for an

---

Corresponding authors

Yuan Li: Tel. +86 0371-67730266; E-mail address: liyuan7626@zzu.edu.cn (Y. Li).

additional 30 min under the same conditions. The pH of the solution was adjusted to 10.0–11.0 with 5.0 mol/L NaOH and stirred for a further 30 min. Thereafter, Then the beaker was sealed with parafilm and left undisturbed for 24 hours to allow sedimentation. The resulting solid was recovered by vacuum filtration, dried at 80 °C for 24 h, and subsequently pyrolyzed at 400 °C (heating rate is 10°C/min) for 2 h under a nitrogen atmosphere. After cooling, the product was ground and sieved through a 35-mesh screen to yield the final MFe@BC material.

Figures:

Fig.S1 VSM diagram of MFe@BC

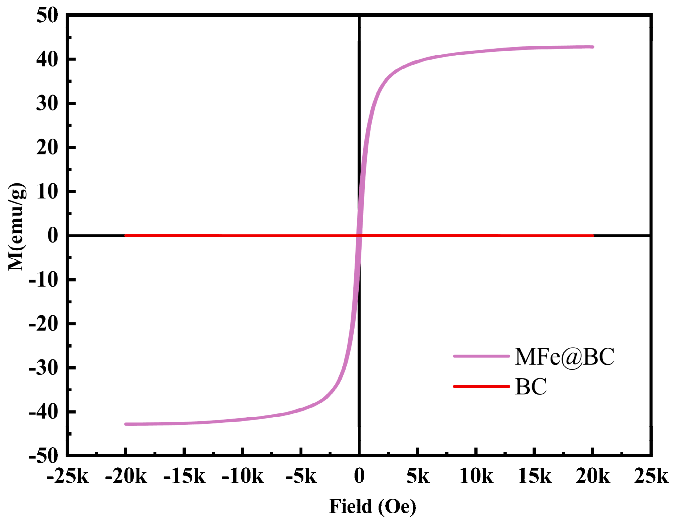

Fig.S1 VSM diagram of MFe@BC

Fig.S2 SEM-EDS and BET of BC

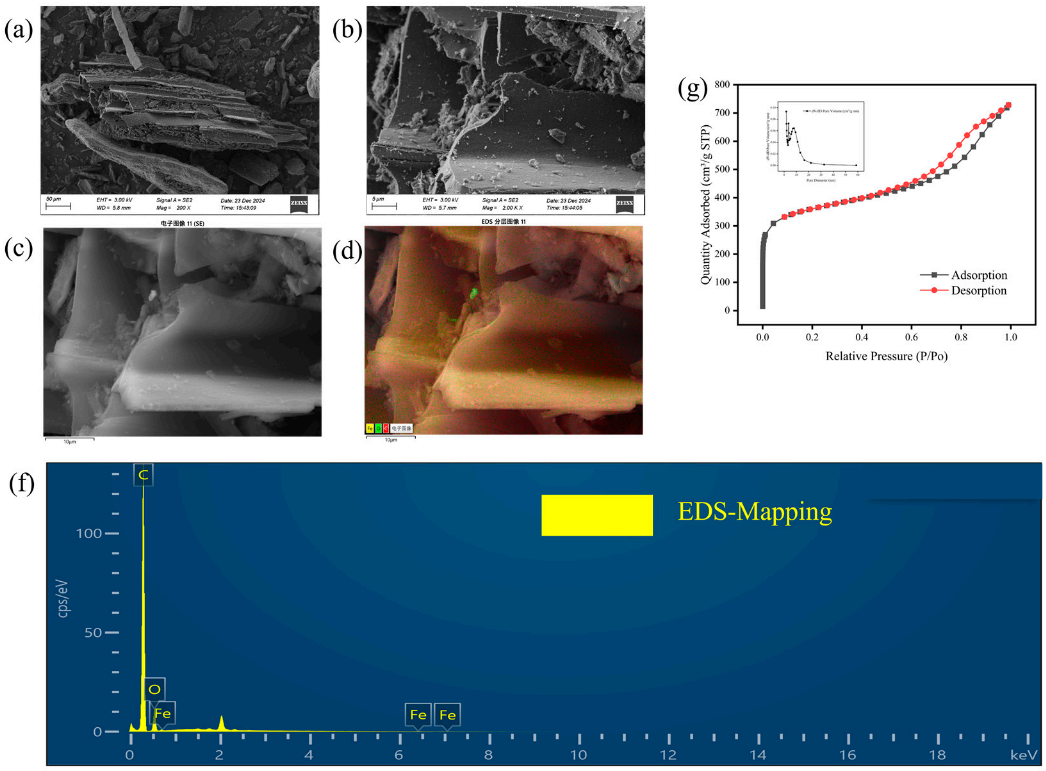

Fig.S2 SEM-EDS and BET of BC

Fig.S3 XPS graphs of C1s, N1s, and O1s of MFe@BC before and after adsorption.

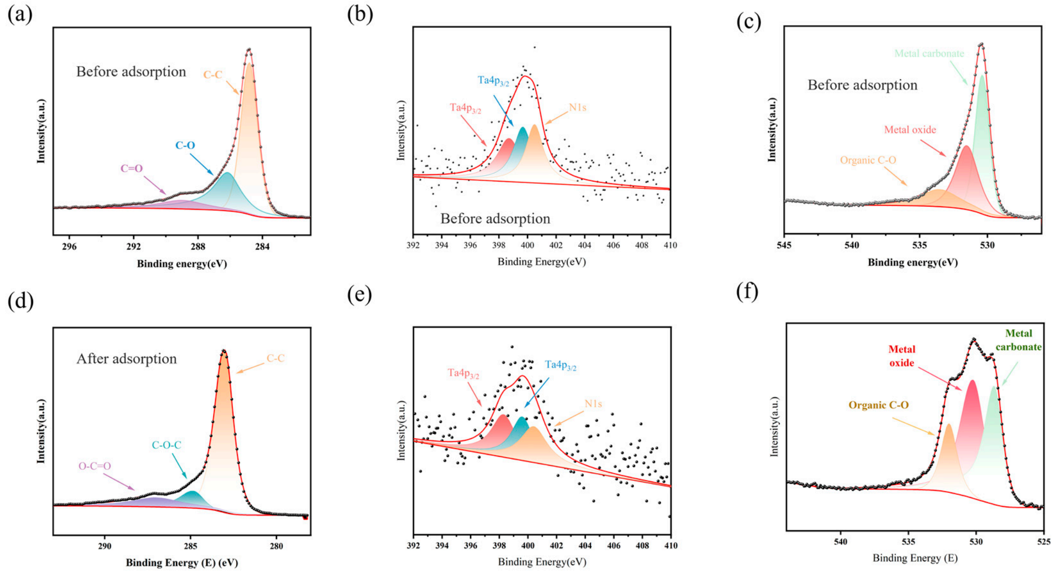

Fig. S3 XPS fine spectra of C1s, N1s, and O1s of MFe@BC before adsorption (a), (b), (c); XPS fine

spectra of C1s, N1s, and O1s of MFe@BC before adsorption (d), (e), (f).

Fig.S4 SEM-EDS of PHBV and MFe@BC after adsorption.

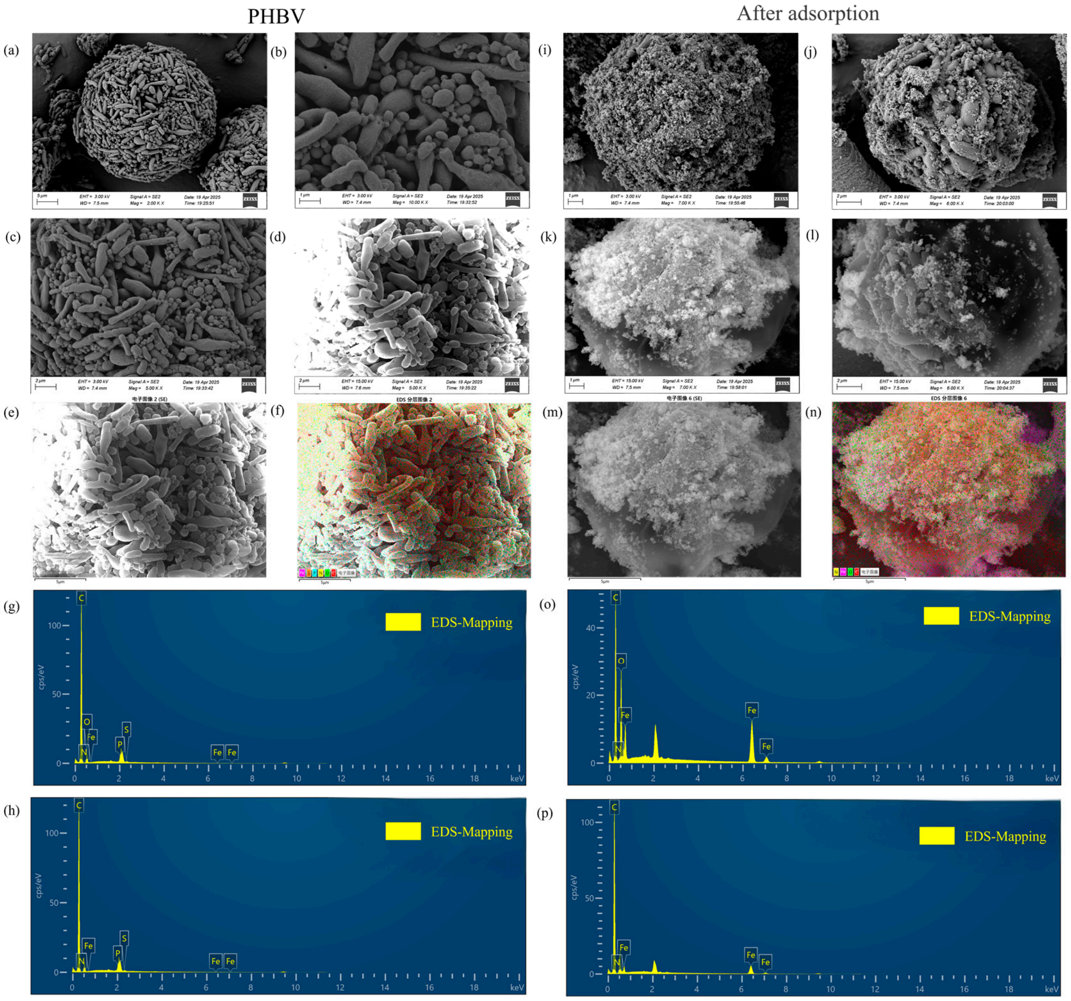

Fig. S4 SEM image showing the surface morphology of PHBV: (a), (b), (c), (d); (e) Electron image of PHBV. (f) Layered image of PHBV. (g), (h) EDS elemental mapping spectrum showing the total distribution of Fe, S, P, N, O, and C. SEM image showing the surface morphology of PHBV: (i), (j), (k), (l); (m) Electron image of PHBV. (n) Layered image of PHBV. (o), (p) EDS elemental mapping spectrum showing the total distribution of Fe, S, P, N, O, and C.

Tables:

Table S1 EDS data of MFe@BC

elemental	line type	wt %	wt % sigma	At %
C	K-line system	33.90	0.10	62.93
O	K-line system	10.74	0.05	14.97
Fe	K-line system	55.36	0.10	22.10
total		100.00		100.00

Table S2 BET data of BC

specific surface area	porosity	average pore size
1,330.6677 m^2/g	0.761157 cm^3/g	6.4046 nm

Table S3 EDS data of BC

elemental	line type	wt %	wt % sigma	At %
C	K-line system	84.58	0.08	88.03
O	K-line system	15.29	0.08	11.94
Fe	K-line system	0.13	0.02	0.03
total		100.00		100.00

Table S4 FTIR of PHBV and FTIR data of MFe@BC after adsorption

	Characteristic peak	Wave number(cm^{-1})
PHBV	C-H	2984.01
	C=O	1732.34
	C-O-C	1284.77

		1184.38
		1134.86
		1096.90
		1055.69
	O-H	3429.64
	C-H	2923.84
MFe@BC after adsorption	C=O	1617.92
	C-O-C	1190.53
	Fe-O	577.94

Table S5 EDS data of PHBV and adsorbed MFe@BC

		elemental	line type	wt %	wt % sigma	At %
		C	K-line system	88.01	0.17	91.02
		N	K-line system	0.00	0.57	0.00
		O	K-line system	11.15	0.16	8.66
	(g)	P	K-line system	0.47	0.04	0.19
		S	K-line system	0.31	0.02	0.12
PHBV		Fe	K-line system	0.05	0.06	0.01
		total		100		100
		C	K-line system	96.08	0.71	97.09
		N	K-line system	2.93	0.72	2.54
	(h)	P	K-line system	0.55	0.04	0.22
		S	K-line system	0.37	0.02	0.14

		Fe	K-line system	0.06	0.07	0.01
		total		100		100
		C	K-line system	74.42	0.17	13.22
		N	K-line system	0.00	0.34	0.00
	(o)	O	K-line system	15.23	0.12	12.98
		Fe	K-line system	10.35	0.09	2.53
MFe@BC after adsorption		total		100.00		100.00
		C	K-line system	84.93	0.41	94.59
		N	K-line system	2.51	0.46	2.40
	(p)	Fe	K-line system	12.56	0.13	3.01
		total		100.00		100.00
